# On-Farm Welfare Assessment of Horses: The Risks of Putting the Cart before the Horse

**DOI:** 10.3390/ani10030371

**Published:** 2020-02-25

**Authors:** Martine Hausberger, Noémie Lerch, Estelle Guilbaud, Mathilde Stomp, Marine Grandgeorge, Séverine Henry, Clémence Lesimple

**Affiliations:** Univ Rennes, Normandie Univ, CNRS, EthoS (Éthologie animale et humaine)-UMR 6552, F-35380 Paimpont, France; martine.hausberger@univ-rennes1.fr (M.H.); noemie.lerch@univ-rennes1.fr (N.L.); est.guilbaud@gmail.com (E.G.); mathilde.stomp@hotmail.fr (M.S.); marine.grandgeorge@univ-rennes1.fr (M.G.); severine.henry@univ-rennes1.fr (S.H.)

**Keywords:** welfare, horse, on-farm assessment, ethology

## Abstract

**Simple Summary:**

The present review examines existing protocols for assessing horse welfare at the farm level. Recent scientific studies converge to promote animal-based rather than resource-based criteria for these assessments because they better represent the actual welfare state of an animal. We examine existing protocols, their strengths and limitations in terms of choice of indicators, sampling methods and scoring and then confront their results with those obtained in different scientific studies. It appears that there is still a need for more research, and we propose lines of thought and practical aspects for improvement. This is highly important in order to avoid under-evaluation of horses’ welfare, which would be deleterious for the discrimination of positive versus welfare-compromising practices. Animals express their internal states through behavioral changes, and the first window we can have into an animal’s mental state of positive or negative well-being is by observing behavioral modifications, which should be the first step. At this stage, in high-income countries, more research has to be developed before disseminating protocols or results to the public. It is important to avoid proposing protocols that rely upon indicators that still need to be refined in order to ensure the reliability of their dissemination.

**Abstract:**

Although the question of animal welfare has been an important source of concern in the scientific community for several decades, many aspects are still under debate. On-farm assessments have to be rapid, acceptable to farmers and safe for both the assessors and animals. They are thus very demanding, with multiple decisions to make, such as the choice of appropriate indicators, sampling methods and scoring. Research has moved from resource-based to animal-based criteria, which reflects the subjective welfare state of an animal rather than relying upon external indices. In the present review, we describe two major (i.e., the most frequently/recently tested or disseminated) protocols: one in low-/middle-income countries, and the other in high-income countries, for on-farm assessments of horses, using animal-based resources; we evaluate their strengths and limitations, and then we compare their results with those obtained by various other studies. We propose lines of improvement, particularly in view of public dissemination, and offer suggestions for further refinement or new protocols. We emphasize the high risks of putting the cart before the horse, i.e., proposing protocols that rely upon indicators and sampling methods that need to be refined, as this could lead to under-evaluation (or less likely over-evaluation) of current welfare problems. Because welfare is a subjective experience, the true representation of an individual’s actual welfare status has to be evaluated by using objective assessment tools (that are validated and have a scientific basis) used by well-trained observers.

## 1. Introduction

Although the question of animal welfare has been a source of concern in the scientific community and of attempts to build communal protocols over the last decades, many aspects are still under debate, such as the validity and reliability of the welfare indicators chosen (e.g., [1]). This is especially the case for on-farm assessments, i.e., evaluation of welfare at a facility population level, for which scientists may have felt forced to answer public concerns rapidly, hence producing suboptimal criteria [2]. The animal welfare scientific community has moved progressively from resource-based toward more animal-based assessments that are in agreement with the fact that welfare state results from subjective experience (e.g., [3]). In this context, on-farm welfare assessment is a very demanding task. It requires valid welfare indicators at the individual level that allow the detection of chronic problems over a short period of time. This means that the sampling method is very important. The number of individuals to assess per site is another point of decision. Finally, the competency of assessors is highly important (e.g., [4,5]). As on-farm assessment is demanding in terms of sample size, many different assessors may be involved, and the question of inter-observer agreement becomes an important issue (e.g., [4]). These aspects are particularly crucial when dissemination to public or professionals is involved, because it is then presumed that (mostly) untrained observers are able to detect behavioral, postural or specific health indicators. This means that proposals should then be based on simple and clearly visible indicators if one wants to limit the risks of over-, or still worse, under-evaluation by owners of their animals’ potentially impaired welfare state. Several recent studies have shown, by comparing owners’ or caretakers’ responses to questionnaires concerning direct observations or manipulations, that even visible behavioral disorders such as stereotypic behaviors can remain unseen and still more so the less specific signs of back problems, one of the most frequent and major welfare issue for riding horses [6,7,8,9]. The fact that resource-based models may seem easier to assess by untrained observers may explain that stakeholders ranked the absence of thirst (i.e., provision of water) and nutrition (i.e., provision of food) as the most important criteria to assess equine welfare, neglecting many other important aspects, in Dalla Costa et al. [10]’s study. This is especially interesting as obesity appears to be a major welfare issue in all recent large-scale studies (e.g., [11,12]), reinforcing the idea that resource-based assessment is not sufficient and can easily become misleading.

The quality of the scientific protocols should thus be reviewed before dissemination. Hawkins et al.’s [13] guide to defining protocols for welfare assessment in laboratory animals proposes a number of recommendations, concerning three aspects: (1) the components of an “ideal” welfare state—physical state (e.g., body condition), physiological state (e.g., hormonal) and psychological state (appropriate range of behaviors in relation to what is known of the species); (2) the competencies of the team members who should be able to recognize a “normal” animal and “ normal” behavior, as well as have the ability to assess and interpret welfare indicators; (3) the selection of appropriate welfare indicators, which should be readily and reliably observable, effective at providing good measures of welfare and practical to carry out. Ideally, a combination of indicators from the three components should be used. Hawkins et al.’s [13] guide insists on the necessary solid knowledge of the species’ behaviors and adaptations as a basis for establishing protocols and suggests multifaceted approaches, combining ethological and physiological/health measures as a way to ensure the validity of the welfare indicators. One recent example comes from horses’ adult play, a behavior highly uncommon under natural conditions, but frequently reported under domestic conditions, and commonly believed to reflect a positive welfare state: measures of oxidative stress (an indicator of compromised health) and back problems, amongst other indicators, showed that, in fact, adult horse “players” may have been trying to cope with their highly compromised welfare under restricted management conditions [14,15]. Thus, while adult play may reflect temporary positive emotions, it certainly cannot be used as a valid welfare indicator, which only a good knowledge of horses’ natural behaviors and a combined behavioral and physiological approach could reveal. 

Beyond the validity of the indicators and team training, the sampling method is another crucial issue: What should be the minimum time of observation? How many repetitions are needed? Behavioral and/or postural modifications related to welfare alterations are based on long-term chronic stress (e.g., stereotypic behaviors), as gastric ulcerations and chronic back pain are based on repeated physiological stress. Thus, the choice of sampling method must combine the constraint of having sufficient data to ensure validity (concerning these long-term problems), while allowing a necessary rapid assessment in the field. In this respect, if the assessment of health problems is relatively easy for trained persons, it is crucial that temporary health alterations be discriminated from chronic (thus, welfare) issues: e.g., is ocular discharge due to a temporary exposure to outside irritants (because the animal is allowed outdoors) or to enduring inappropriate housing conditions [16]? Moreover, is irregular locomotion due to a temporary event (e.g., incident outdoors) or to a chronic problem (back pain)/working conditions (see also [1])? Classical ethological studies such as Altmann’s [17] have contributed largely to the awareness of the scientific community concerning the difficulties to choose the right sampling method according to the type of behavior/activity considered. It is well-known that welfare issues have consequences on time-budgets (i.e., time spent in given activities), behavioral repertoires (presence/absence of behaviors) and behavioral/postural modifications (repeated behaviors, stereotypies) (e.g., [18,19]). Such modifications are the expression of both psychological and physiological problems, as both are clearly related (e.g., [20]). Ideally, therefore, the scientific team developing and testing a protocol needs, in addition to being trained to assess health issues, to have both a solid background in ethology and knowledge of the field situation to ensure feasibility, so that the psychological part of welfare can be taken into account. For on-farm welfare assessments, it is very important then that a thorough training is provided to stakeholders, veterinarians or caretakers who will apply the chosen protocol and raise public awareness of these issues. Although the opportunity to access a “reference” population of a domestic species [20], i.e., “normal” animals with “normal” behaviors, may be rare, this assessment is necessary to constitute the “reference” (as suggested by Hawkins [13]) before examining a population with a high prevalence of welfare problems. It has been shown that over-exposure to “abnormal” syndromes lead horse caretakers to overlook major expressions of welfare problems [6], a problem also identified in the community of human health-care professionals (e.g., [6,21]).

Finally, all persons working on animal welfare agree that on-farm assessments must be rapid, acceptable by professionals, thus requiring as little handling as possible, and be safe for both assessors and animals (see below). In this article, the authors introduce an up-to-date review of the literature, in order to assess the reliability and validity of protocols proposed to assess welfare in working equids. Special consideration is given to physiological and psychological aspects. The review is divided into two parts: (1) detailed descriptions of two major (the most recently used and disseminated) protocols proposed for on-farm assessments of horses, using animal-based resources, looking at their strengths and limitations; and (2) an examination of potential problems in terms of indicators or sampling methods, and suggestions for improvement especially in view of public dissemination, particularly by putting more emphasis on behavioral expressions of welfare. Only direct observational studies are considered here. The term “on-farm assessment” has been repeatedly used in different studies of working horses: thus, although it is not commonly used for such animals, the term “farm” will be used here as a general term for horse facilities (riding centers, private owners, breeding stables, etc.).

## 2. Examining Some Current Standardized Protocols

In this part of the paper, we describe two protocols of on-farm welfare assessment of horses that rely upon animal-based resources:

One, SEBWAT (for “Standardised Equine Based Welfare Assessment Tool”, originally WEWA, (“Working Equids Welfare Assessment”) was developed for evaluating the welfare of working equids in low-to-middle-income countries (LMICs) [22]. In this protocol, the animal is in its working environment, and the aim of the developers is not necessarily to have an exhaustive view of the welfare conditions. The other, AWIN, was developed for adult horses living in single stalls, in high-income countries [23].

After having described these protocols, we review the results of the studies using these protocols, and discuss the strengths and limitations of each protocol.

### 2.1. SEBWAT: Standardised Equine Based Welfare Assessment Tool

This welfare assessment system has been developed for providing the Brooke Hospital for Animals charity with a methodology that could help provide treatment for equines working in low-to-middle-income countries (LMICs) and advise owners for better management practices. Thus, the purpose of the tool was to provide an overview of the general welfare condition of working equine animals, both individually and at a group level. The choice was to use animal-based (health and behavior) inputs as an alternative to resource-based inputs, because they reflect more of an animal’s perception of a situation but also because the primary aim was to examine the equids in their working location [24] The requirement was that the protocol should be suitable for practical use under field conditions, performed in less than 10 min and, for most measures, without touching the animal. The tool has been applied in practice by a non-governmental organization (NGO) since 2012, across LMICs. Sommerville et al. [25] described the revision of the tool into SEBWAT in 2018. The tool has been used more than 71,000 times, in 11 countries, since its development.

#### 2.1.1. The SEBWAT Protocol

First, a list of health and behavior parameters was established by 15 experts, with the aim of developing a tool based on direct observations. Forty animal-based measures of relevance to working equine welfare and some additional data identifiers have been chosen. Indicators of health were related to three issues: wounds, lameness and body condition, to which heat stress was added later. Some aspects required intervention: examining the sole surface of the hoof on the right forefoot or gait abnormality, and animals are restrained by using a head collar or halter by an assistant experienced in handling equids. Welfare measures are concentrated around evaluations of the horse’s behavior toward a human approach or handling, for which novel tests were developed. A checklist was categorized into observations of behavior, general health parameters, body condition, limb disorders and lesions of skin or deeper tissues. In the field, observers work in pairs, with one person making observations and the other recording the results, but roles are rotated every five animals to ensure concentration. The assessors are trained during 6-to-10-day sessions and receive a handbook of photographs and descriptions [24]. The protocol ensures an assessment lasting 10 min at most and is performed at the animal’s place of work or housing. When at work, tack was taken off when possible.

Behavioral measures include the animal‘s general alertness (i.e., responsiveness to environmental stimuli) at a distance for 10 s, reaction (none, friendly and avoidance/aggression) to a human (assessor and then owner) approach and walking alongside the animal’s body, and to the “chin test”(hand in firm contact under the chin). Assessment of physical health, made after the behavioral evaluations, includes lips and head, and any eye abnormalities, body condition score (BCS on a 5-point scale), recording fecal soiling, skin tent duration on the animal’s neck and any behavioral signs of heat stress [26,27,28], noting ectoparasites and lesions over the body and limbs, visible swellings of the flexor tendon or fetlock joints and hoof health, including picking up the right fore-foot to examine the sole surface. Finally, gait is assessed by watching the animal walk as the owner leads it away and back.

Training of assessors was designed to ensure a good level of standardization (minimum of 80% in the standardization test) and received theoretical knowledge. Although a detailed guide is provided, it is underlined that assessors cannot rely only upon these guidance notes and must have practical training.

#### 2.1.2. Results from Studies Using SEBWAT

A first study including 2071 horses of various ages and breeds in five LMCIs countries [22] showed that horses responded mostly by no response (55%) or negative (avoidance/aggression) responses (26%) to human approach, 70% were thin, few (8%) had small health problems (ectoparasites, poor coat condition), but half of the horses had an increased skin tent duration, revealing some dehydration. Ninety percent showed some gait abnormalities, and 80% had an abnormal sole surface. The lack of responsiveness to environment/handling was correlated with a poor body condition score, lesions of skin or deeper tissues and abnormal gait.

Burn et al. [29], in a follow-up study, observed 4504 horses in nine developing countries, involving 42 trained observers, who were mostly veterinarians. Behaviors included the animals’ general alertness, and their responses to four human-interaction tests, using an unfamiliar observer as the human stimulus. Avoidance behaviors correlated significantly with each other across the human-interaction tests, with 21% of the animals avoiding the observer, but they showed no associations with equipment injuries. Over 13% of the horses appeared ‘apathetic’, i.e., unresponsive to their environment and human approach. Measures of unresponsiveness correlated with each other across the different tests and were associated with poor body condition, abnormal mucous membrane color, fecal soiling, eye abnormalities, more severe wounds and older age. The authors suggest that working equids in poor physical health show an unresponsive behavioral profile, consistent with illness, exhaustion, chronic pain or a depression-like state. Since then, many studies have been performed in different developing countries, and all reach more or less the same conclusions in terms of prevalence of welfare problems (e.g., [30,31]).

Popescu and Diugan [32] adapted the protocol for a study of working horses in Romania, involving 715 horses of various ages, sexes and breeds. Two assessors were involved who had been trained until they reached at least 80% inter-assessor agreement. As for the initial protocol, a short questionnaire was given to the owners for descriptive indicators (horse’s sex, age and type of work), but there were also questions such as access to free exercise (i.e., none, limited: at least 1 h/day, 6 months/year, unlimited: at least 9 months/year with access to an area large enough to allow rolling and performing different gaits). General alertness was observed from a distance of 3–5 m for 60 s (instead of 10 s in the initial protocol) and unresponsiveness to their environment (no eyes open, movements of ears, head or tail) was qualified as « depressed » (head lowered, eyes half closed, reduced movements of ears, tail and skin) rather than apathetic. They applied Burn et al.’s [29] three human–horse relationship (HHR) tests that are performed by an assessor and the owner: In the first test, they approach the horse, stop 30 cm from its body and record the horse’s reactions when they stop; in the second test, they walk slowly all around the horse’s body and record any sign of attention; and in the last test, they try to touch the horse’s chin. They also had 19 health-related indicators that were partly issued or modified from Burn et al. [28], according to the field conditions and the results of a preliminary study. Overall, half of the horses responded to the assessor’s approach with indifference, while a similar proportion showed « negative » responses (avoidance/fear or aggression). Most of them (64%) showed avoidance/fear when the assessor walked along their side or touched their chin (48%). They showed more indifference overall when their owner did the tests, apart from the chin test. Almost 8% of them showed aggressiveness in this test both towards the assessor and the owner, and there was a low consistency of horses’ responses between tests. Some correlations appeared between behavioral and health measures: the 3% of the horses showing a depressed state were more prone to have deep tissue lesions, the 25% of horses with swollen tendons/joints were more likely to be aggressive in the three human–horse tests and barefoot or adequately shoed horses were more likely to be indifferent or friendly in these same tests. Finally, most (84%) of the studied horses had no access to free exercise, and they were those showing the less friendly response toward the assessor in the HHR tests. In a more recent study of 1482 horses, the same authors added two parameters (one questionnaire-based: number of times teeth had been examined and one observational: hair quality at mane/tail: broken, matted and dirty versus complete, clean and shining), as well as an overall score calculated by summing all measures [33]. Their results indicate that working horses (draught: agricultural works and carrying of loads with carts) showed more indifference toward humans than breeding horses, and that most (up to 60%) horses showed negative responses (avoidance/fear or aggressive reactions) in the chin test. Overall, the working horses had more welfare alterations, and in particular, lesions/wounds at harness points (up to 50% of the horses had inappropriate shoeing, etc.) than the breeding horses.

In conclusion, overall, these studies show some fairly convergent findings: a low proportion of the working horses tested showed friendly reactions to humans (15% in Burn et al. [29], 19% in Pritchard et al. [24] and 24% in Popescu and Diugan [32,33], but see Tadich et al. [34], with up to 64% friendly horses). The prevalence of avoidance/fearfulness and aggressiveness increased largely in the walk-beside test, and the highest prevalence of aggressive reactions was for the chin contact test in all studies, confirming that horses react more clearly to more “invasive” approaches [35]. Unresponsiveness (to environment and in the HHR tests) correlated with several health impairments in the two first studies, but this was less clear in the two others. This may of course be due to the differences between the populations studied in terms of types of horses, of work and general management (housing, feeding, etc.), but also to discrepancies in the definitions of “unresponsiveness”. Burn et al. [29] and Pritchard et al. [24] studies showed that unresponsiveness was correlated between general alertness and response to humans’ approach whereas no such link was foundbetween both in Popescu and Diugan [32,33] studies, where very few horses appeared “depressed”. The high proportion of horses showing “indifference” in the HHR tests was interpreted as being due to horses being used to the approach and proximity of different people, and thus as a neutral response by Popescu and Diugan [33], whereas “unresponsiveness” to humans for Pritchard et al. [24] and Burn et al. [29] reflected compromised welfare. It is worth noting that Popescu and Diugan’s study [32] showed that the working horses were more indifferent and had also more welfare alterations than the breeding horses, suggesting that “indifference” may not be that neutral.

#### 2.1.3. Strengths and Limitations

Strengths: This protocol is considered by its developers to have several strengths, as it provides the following: an overview of the general welfare state of an animal and a population, and a reliable representation of an animal’s real welfare state (and extent of coping), thanks to the animal-based measures; the formal training of assessors aims to reduce inter-observer variability; the explicit definitions of scoring criteria reduces observer bias, allows quick assessment without disturbing activities much, involves little contact with the animal. It is considered that, in practice, through the process of closely examining an animal during welfare assessment, the causes of the welfare problems observed can often be identified while collecting the data. To these identified advantages, one can add the multifaceted approach that allows to demonstrate that indeed horses’ reactions to humans, although not specific to welfare issues, may reflect them and draw attention toward further scrutiny of other indicators. Although aggressiveness is known to be a possible indicator of pain (e.g., [36,37]), its interest as part of a welfare assessment had not been demonstrated before. The importance of alertness versus unresponsiveness/apathy/indifference is also well demonstrated by these studies while being questioned [38] and confirmed by other welfare studies that also show correlations between unresponsiveness/depressive states and physiological/health disorders [20,39,40].

Limitations: The protocol promoters identified some limitations: requirement for training and standardization (only practically trained assessors can use it properly); requirement for periodic standardization of trained welfare assessors to maintain a low level of intra- and inter-observer variability; because of the scoring system, results must be translated into laymen’s terms for untrained audiences; time is required for data handling subsequent to the period of data collection. Some complementary limitations can be mentioned. For example, not all measurements show the same inter-observer reliability. Burn et al. [4] showed that agreement was low (<75%, Kappa < 0.4) for eye abnormalities, horn quality, and lesions at fetlock and ribs, as well as coat condition (i.e., difficult cut off point between healthy, dull or poor conditions). Intra-observer variability was also observed for the human–horse tests (repetition effect?), while the inter-observer reliability was high for the approach and horses’ chin tests. Hence, some measures could be more useful than others or descriptions should be refined. Some other aspects, such as the indicators chosen, would require further descriptions and thought. For example, using the neck shape for assessing body condition, as done in some studies (e.g., [29]) using this protocol, may be misleading, as a hollow neck may be due to working conditions more than actual body condition [41,42]. « Unresponsiveness », « apathy » and « depression » would deserve to be redefined in view of recent reports that describe these terms with more objective criteria (e.g., time without absence of movement, eye blinking [40]). As mentioned by Burn et al. [29], it can be difficult for an insufficiently trained observer to discriminate these states from resting standing, especially if restrained to times when the horse has its eyes half closed, as in Popescu and Diugan’s [33] study, whereas in Fureix et al.’s [40] study, “depressed” horses had a fixed glance with open eyes (see also [38]). This could explain partly the low proportion of such horses in their population despite clear welfare alterations. The counterpart “alertness” could be redefined as “attentional engagement” and estimated by sampling the time spent in observation of their environment [43]. “Alertness” may be interpreted as “vigilance”, leading observers to put calm attention and “alarm” postures (that may also reflect a higher emotionality due to welfare alterations) in the same category. Because assessments are mostly performed at working sites while the animals are restrained, the range of expressions of their welfare state could be restricted: for example, there is no mention of stereotypic behaviors in these studies, which, no doubt, would have been added to the protocol if they had occurred frequently during the assessments. Although these behaviors can be observed during work in ridden horses [44], they remain much more infrequent than when the same horses are observed in their usual housing conditions. Finally, while the alertness of a horse is assessed within 10 (or at most 60) seconds from a distance, one could wonder whether sampling over a longer period of time would not be more reliable. There is no clear mention of having tested the “ideal” sampling method: one overall assessment over 10 s or scan sampling over a few minutes, for example [43]?

#### 2.1.4. Conclusion

SEBWAT is a very interesting protocol that has helped draw attention to welfare indicators that were not explicitly developed in earlier welfare studies, such as responsiveness to the human and non-human environment, i.e., attentional engagement. It fulfils the initial aim, which was to assess the welfare of working equids and shows that, with some clear-cut standardized observations, it is possible to have a multifaceted view of an animal’s welfare state. All these studies, largely based, on the behavioral side, on human–horse relationship tests, converge to show a low proportion of positive responses of working horses to humans, which is the only « positive indicator » present in the protocol. Some limitations are the precision with which some indicators and the rationale for the sampling method are described and also, for a more global view of animals’ expressions of their welfare state, observations outside work. This is indeed a limitation for identifying the whole range of possible causes for the compromised welfare observed. Finally, this is not a protocol designed for public dissemination but can fulfill its educational role when trained staff give information to equid owners. Curiously, this protocol has not been used much in on-farm assessment studies of horse welfare in developed countries, which would allow interesting comparisons.

### 2.2. AWIN Welfare Assessment Protocol for Horses

The AWIN protocol was developed through a 7^th^ Framework European program (1^st^ May 2011-30st April 2015), with the aim of developing and disseminating animal-based welfare indicators, including indicators of pain. A panel of academic scientists was involved as partners or collaborators to work on a protocol of on-farm welfare assessment with animal-based indicators for five domestic species (sheep, goats, horses, donkeys and turkeys). We mention here only the protocol developed for horses. At the present stage, it is proposed for horses over five years old and housed in single stalls, where the assessment takes place [45]. It is specified that proper training and adequate knowledge are essential to apply this protocol, but the application that was developed for this protocol is apparently designed for a larger public use.

#### 2.2.1. The Protocol

The framework of the protocol [23] was the Welfare Quality^®^ concept of “5 freedoms” that have given rise to four principles and 12 criteria: good feeding (i.e., absence of thirst and hunger); good housing (comfort around resting, thermal comfort and ease of movement), good health (absence of injuries, disease and pain) and appropriate behavior (expression of social behavior, other behaviors, good human–animal relationship and positive emotional state) (see [46]). The AWIN work started with an examination of the database on animal-based welfare indicators since 1980, including peer-reviewed publications, proceedings and abstracts in English, and to place them within the Welfare Quality^®^ frame. Experts examined this database, with the aim of identifying which indicators were valid (in accordance with what they were supposed to measure), reliable (repeatability in time, within and between assessors) and feasible (practical likelihood of using this indicator during on-farm inspection). Gaps in knowledge were also identified in order to promote research to fill them. The developers identified 49 indicators based on 54 papers but considered that only a few met the criteria for inclusion in the protocol at that stage. Despite the primary aim of concentrating on animal-based resources, for some of the principles or criteria, it was considered that there were no valid or reliable indicators and preference was then given to resource-based indicators (e.g., water availability and cleanliness, stall size, possibility of social contact, information on turnout and work). In all, 24 indicators were retained: (a) 3 for the principle “good feeding”: body condition score (a 5-point scale), water availability and a bucket test; (b) 3 for “good housing”: bedding, box dimensions and exercise; (c) 12 for “good health”: integumentary alterations, swollen joints, lameness, prolapse, hair coat condition, discharges, consistency of manure, abnormal breathing, coughing, horse grimace scale, signs of hoof neglect and lesions at mouth corners; (d) 5 for “good behavior”: stereotypies, fear test, human-animal relationship tests and qualitative behavior assessment. A detailed description is given in a guide booklet. According to the indicator, either a presence/absence or graded response can be given: for example, presence/absence of water; dirty, partially dirty or clean water. For some resource-based indicators, reference is the Swiss Animal Welfare Ordinance (stall size), responses from the primary caretaker (e.g., time out of stall) or self-assessment (e.g., litter cleanliness). Moreover, in the absence of valid direct animal-based indicators of positive welfare, the authors chose to use the “Qualitative Behavior Assessment” (QBA) [47], based on humans’ appreciations of the animal’s style of behaving, using descriptors (e.g., “relaxed” versus “tense”). In the present case, the assessor observes a horse from outside its stall for 30 s, then enters and scratches the horse’s withers for 30 s and then evaluates the animal’s style of behavior for 30 s, considered as one integrative assessment over the whole observation period (30 s before and after scratching). Scores of intensity and duration of behavior are given on a “visual analogue scale”. Most measures do not require the observer to touch the animal and can be performed outside the stall, but some health measures require going close to the animal.

Direct sampling methods and measures of behaviors vary according to indicator: 1 min observation sessions (or indirect signs) for stereotypies (crib-biting, weaving, head nodding and wood chewing) and one measure of presence/absence; measures of latency to approach or re-approach a novel object (a green plastic bottle filled with little stones first hanging then dropping in the stall) in the fear test; presence/absence of avoidance in the avoidance test (human approaching with an arm raised at 45° from chest), 20 s observation of human-directed behaviors in the voluntary approach test where the assessor behaves as if he/she is going to open the stall door.

Propositions are given concerning the number of horses to be assessed according to the farm horse population for a first-level approach, and if wished, all horses from the farm for a second-level approach. The first-level approach requires no handling, whereas the second-level approach is more complete and includes entering the box for a “forced human approach test” and a fear test, plus asking a handler to move the horse to assess potential lameness.

#### 2.2.2. Results of Studies Using AWIN for Horses

One study was especially designed to test the protocol, and it was performed by the two research groups that had proposed the AWIN protocol for horses [10]. They applied it to populations in their respective countries (Italy and Germany), examining 355 sport and leisure horses of various ages, sexes and riding disciplines, stabled in 40 facilities (single stall housing). Three trained assessors, all veterinarians, were involved. The AWIN Horse app was used to collect, store and send data to a common server. Between 5 and 25 min were required per horse.

In terms of resource-based indicators, automatic drinkers provided water in more than 90% of the cases, but in almost half the cases, the water was partially or really dirty. Bedding provided was in the majority of cases assessed as sufficient (81% including rubber mats) and clean according to the AWIN criteria and box dimensions were scored as satisfactory in 68.6% of the cases. In total, 22% of the horses had no social contact at all, 39% had only visual contact and the other 39% had some possibilities for either sniffing or touching another horse. In terms of management-based indicators, about half the horses had the possibility to exercise (freely or ridden) on a daily basis, 28% once to four times a week, while 9.3% had no opportunities to leave their box at all.

The results of the animal-based indicators show that a large majority of the horses were in normal or excessive body condition (87%), with 32% being overweight, but that most horses presented no health problems and the most frequent integument alterations were alopecia, followed by superficial skin lesions and swellings. Some measures appeared difficult to obtain: lesions at mouth corners when handlers were not available to hold the horse’s head, lameness for 14.4% of horses and manure samples, as most of the times boxes were clean at the time of the inspection and no feces were present. The prevalence of stereotypic behaviors was 19% and was significantly related to the reduced possibility of social contact (χ² test, *p* = 0.001). During the human–horse relationship tests, 79% of the horses showed positive signs in the Forced Human Approach (FHA) test, while only 2% to 3% showed negative signs. Overall, a minority of horses showed a negative relationship to humans, and 70% did not show any avoidance in the distance avoidance test. However, testing avoidance distance to a human approaching the stall door was not possible in 23.3% of the cases, mostly when horses were inattentive to the human’s presence. Results of the fear tests are not clearly mentioned.

Identified welfare issues were overall obesity, unsatisfactory stall dimensions, long periods of confinement and lack of social interactions. Unresponsiveness to humans (even after clicking three times with the tongue) could be observed forsome horses, and this was interpreted as a neutral response. The authors mention that further scientific research is needed and that the protocol will be updated for use in different conditions in the light of new scientific knowledge.

Another study, with the primary aim to test inter-observer reliability (see below) took place in Germany on 10 farms and included a total of 435 horses of various ages, sexes and breeds, although they were mostly warmbloods [48]. Breeding, leisure and sport horses were involved, and housing varied from single stall to groups housed outdoors. Two assessors, a veterinarian and an agricultural scientist, performed the evaluation. The results of the animal-based indicators show that over 98% of the horses presented no stereotypic behavior, good hair condition and normal breathing; over 90% showed no avoidance; and 70% were friendly in the tests with humans. No aggressive behavior was observed. Overall, no major health or behavior problems seemed to arise. The authors mention that the protocol was feasible in terms of time, as no more than one day at the most was needed to assess horses in large farms with 100 or more horses.

In conclusion, the results of both studies based on the application of the AWIN protocol are quite congruent: They indicate a low prevalence of behavioral problems and good health conditions, especially the second study [48]. The first study confirms the prevalence of obesity of horses in developed countries. Since “unresponsiveness” is not part of the indicators chosen, it is not or only anecdotally mentioned. Feasibility appeared high, as time criteria for rapid assessment are mentioned as being met in both studies.

#### 2.2.3. Strengths and Limitations

Strengths: The large interest of this protocol is of course to be applicable on large populations of horses and in their home environment, allowing an evaluation based on a variety of indicators. It is at that stage the most exhaustive protocol proposed, which moreover allows evaluation in a limited time window. A guide gives explanations of the different measures, and their application allows observers to enter the data on a central server. Although restricted to single-housed horses, extension for assessment of group-housed horses is underway. Czycholl et al.’s [48] study, with two observers assessing the same horses at the same time, showed that inter-observer reliability was overall good, especially when using a kappa agreement coefficient corrected for both bias and distribution of data, the Prevalence Adjusted Bias Adjusted Kappa, PABAK. Kappa calculations yielded scores between 0.35 and 1 (ex: stall size), the highest scores being logically for resource-based data (e.g., stall size and exercise). This is in agreement with the results of a pilot experimental study, which mentioned that difficulties could be encountered in assessing the horse grimace scale [49].

Limitations: There are, nevertheless, certain limitations that should be taken into account for the improvement planned by the protocol promoters.

Training and dissemination: Although it is indicated that only trained persons with knowledge should use the protocol, it is not clear how training is performed (i.e., practical as for SEBWAT? through videos or photos?), or what type of knowledge is expected. Moreover, dissemination has already been large, meaning that the general public could start wanting to use it despite the authors’ indications that some expertise is needed. The lack of precisions on this expertise opens the possibility that any horse owner feels expert. Moreover, dissemination may be too early in view of the need for revisiting some aspects (see further).

Choice of indicators: (a) *Animal-based indicators:* Although it is said that a review of existing indicators had been made and only valid indicators had been retained [23], a few problems remain concerning these choices. The literature review included abstracts and proceedings that are not reliable scientific sources, since they are not peer-reviewed, but also some indicators proposed by other authors have not been investigated, such as alertness [29], depression/unresponsiveness [40] and neck shape [41], as some examples where validity has been shown through correlates between behavior or posture and abnormalities of physiological measures. Few of the indicators chosen have been validated by crossing behavioral and health or physiological measures, an analysis that would be expected for welfare indicators. Only a restricted number of stereotypic behaviors is mentioned, which may lead the observers to believe that horses performing windsucking, repetitive door knocking, box walking, tongue play and other abnormal repetitive behaviors [50] are just “normal” horses performing “normal” behaviors. The rationale for the choice and protocol of the human–horse relationship tests would deserve further explanation. Dalla Costa et al. [51] describe the tests, but they do not really explain why they chose these particular tests rather than any of the others commonly used by horse researchers (see review in Hausberger et al. [52]), nor their precise modalities (e.g., have the arm at 45° from chest in the avoidance test), beyond applying the avoidance test because it was done on other species. Some of Burn et al.’s [29] tests have been used for donkeys, but none of those developed for horses by this same team was tested. Burn et al. [29] and Pritchard et al. [24], for example, have shown clear correlations between horses’ reactions (or lack of reaction) to the “chin test” and different health disorders; Fureix et al. [37] showed that the reactions of horses to different HHR tests were correlated with back disorders. However, there has been no such validation of the HHR AWIN tests in terms of welfare evaluation: horses in facilities classified as suboptimal (according to official reports) in terms of human–horse relationships, proved more negative in the tests, but potential correlations with health or behavioral problems were not investigated [51]. Further investigations at the individual level are thus still needed. The same is true for QBA. Moreover, although studies report correlations between the results of QBA and some behavioral tests, the question of the validity of QBA remains under question [48,53], as for all measures entirely based on subjective assessments. As mentioned above, being playful does not necessarily mean being happy for an adult horse [13], and human representations of the significance of behaviors may be influenced by a variety of factors, such as culture, access to a reference population and personal experience (e.g., [6]). The relationship between welfare indicators and results of the QBA was not straightforward for donkeys and hence would deserve further investigation [54], while validation has not been tested for adult horses.

While hair coat assessment has been validated for goats, a “rough coat” being associated with thinness and lower quality in hair mineral assays [55], it has, to our knowledge, never been validated (i.e., clearly correlated with physiological/health or behavioral measures) for horses, even though Pritchard et al. [24] and Czycholl et al. [48] found some inter-observer reliability. This measure presents a specific problem for horses as horses that are allowed time for free exercise outdoors may not have “shiny glossy hair” when back in their stall, as horses readily roll in mud when possible. Popescu and Diugan [33] assessed breeding mares (which were allowed free exercise outdoors every day) as having a lower hair coat quality than breeding stallions (which were kept permanently tethered indoors) when observed in the same facilities in winter. Should then the recommendation be that horses are kept tethered indoors? Obviously, although hair coat quality is a good candidate for representing health quality, in the absence of precise criteria, its use as an indicator for horses may be misleading. Also as mentioned for SEBWAT, some ocular discharges may be due to temporary turn-out in windy weather. (b) *Resource-based indicators:* Stall size is assessed in reference to the Swiss legislation, but to what extent does this legislation reflect proper assessments of the relationship between stall size and welfare state? Bedding is assessed according to its cleanliness independently of its type despite the extensive demonstrations in the literature of the importance of bedding type for horse welfare: does any study compare horse indicators of welfare with straw bedding, even not completely clean with that observed in stalls with clean paper shaving or rubber mat? How was this validated for inclusion in the protocol? Exercise includes both free movement (e.g., paddock) and work (e.g., lunging and riding), whereas much evidence shows that free exercise has a very different effect on horse welfare from working exercise and is actively chosen by horses when given the opportunity [56,57]. Whisher et al. [58] have shown that cribbing may increase with increasing working time, whereas stereotypic behaviors overall diminish when horses have access to free exercise (e.g., [59]).

Sampling and scoring methods: The scores to be given remain unclear for some parts. For example, there is no intermediate between a dirty and clean bedding, healthy and unhealthy coat, thick and fluid nasal discharge. Stereotypies are sampled through a one-minute session of observation without any explanation for this time span, which is very short. Stereotypic behaviors are not produced continuously and are more frequent before meals (e.g., [50]). No mention is given of when these observations should be done, which means that two farms or individuals may not be assessed in a comparable way. There is no clear mention of how to measure the reactions to humans in the tests: is it the first reaction (as in [60]) or an overall assessment over the procedure time (as in [61]) (but then the assessment is subjective)? Czycholl et al. [48] mention that scoring may be difficult when horses for example are successively indifferent, approach and then withdraw during testing. Mere threats (e.g., turning ears back while staying at the same place) are overseen.

#### 2.2.4. Conclusion

The AWIN protocol for horses is based on the dynamic research by the concerned research group and is a very interesting concept. However, because it was developed rapidly and aimed to have an exhaustive representation of horse welfare, assessed in a minimum of time, it necessarily gives rise to shortcuts and difficulties in terms of having the optimal sampling and scoring methods. At this stage, it seems that many aspects would merit further investigation before the protocol is largely adopted and distributed. Although based on the Welfare Quality^®^ concept, most indicators differ, in terms of type or measure, from those proposed by the “Welfare Monitoring System–Assessment for Horses” [62] that identifies, for example, eight different abnormal behaviors (instead of three here), and puts more emphasis on aspects like time spent in free exercise. This system is very detailed, although lacking many of the animal-based indicators discovered since then. Despite this, the Welfare Quality monitoring system includes many more items than AWIN and also gives an almost equal importance to animal-based and resource-based indicators. This may explain that the developers of AWIN, like developers of other protocols tried to obtain a “lighter” version to increase feasibility [16,63].

## 3. Accuracy of On-Farm Welfare Assessments and Suggestions for Improvement

### 3.1. How Accurate Are On-Farm Welfare Assessments Currently?

Thus, both the SEBWAT and the AWIN protocols, applied in low-income and high-income countries, respectively, lead to quite opposite, but somehow culturally expected patterns. Most studies with SEBWAT in low-income countries reveal a high prevalence of compromised welfare signs, whereas the first studies performed with AWIN in high-income countries indicate overall good welfare. Earlier studies, however, showed that prevalence of welfare issues can be comparable or even higher in high-income countries (e.g., Lesimple et al. [59] reported 68% of equipment-related lesions on 306 riding school horses versus Burn et al. [29], who reported 32% on working horses in low-income countries). Lameness is described for 14% of the 2954 horses examined by Visser et al. [16], but for around 2% in the AWIN studies. Thus, differences between the conditions offered in both types of countries and the cultural attitude of owners toward horses [31] is only one amongst different possible explanations. The high score of “good welfare” in the AWIN studies is also surprising, given the way of life of the assessed animals. As mentioned by the authors, confinement and lack of social interactions, which are known factors of alterations of welfare, were predominant in the populations studied [10,23,49]. It is therefore surprising that behavioral signs of altered welfare were so limited, especially as obviously a number of stalls had no straw bedding (e.g., [64,65]). Despite these restricted conditions, only 19% and less than 1%, respectively, of the horses observed presented stereotypies. Other observational studies of horses living in similar conditions have led to much higher numbers: 37% to 65% of more than 400 riding school horses [59,66]; 28% of 114 stall housed Arabian broodmares [67]. Another striking aspect when comparing the prevalence of comparable data between the two above-described protocols is the huge discrepancy in terms of human–horse relationship, with the majority of horses showing negative behaviors or unresponsiveness in most SEBWAT studies, and the majority of horses showing friendly behaviors in the AWIN assessments. This last result, with only 2.5% of horses showing negative reactions in the human–horse tests, is surprising as all other behavioral studies performed on single-stall-housed horses in high-income countries showed a prevalence of 30% to 70% (224 sport horses [60]), 34% (306 riding school horses [59]), 71% (59 riding school horses [35]). Of course, again, countries differed, and probably practices did somewhat too, but the deleterious effects of single-stall housing have been described in many countries over the world (United Kingdom [68]; Germany [69]; Netherlands [70]; France [59]; Denmark [71]; USA [72]; Brazil [73]). Differences in terms of measures, sampling and scoring are more likely to explain these discrepancies. Because we need to raise public awareness concerning horse welfare and the factors involved, the higher risk is to underestimate the prevalence of welfare issues, which is why the “optimistic” outcome of the AWIN studies is worrying.

Indeed, one other possibility is that the AWIN protocol as it stands currently, because it had to be developed within a limited time span (the European 7^th^ Framework) and because of the constraints in time imposed by the principle of on-farm assessment may not have reached yet its ambitious goal, which is to give a very complete view of the welfare state of domestic horses in current domestic European husbandry. Maybe we are just not ready for such an ambitious evaluation yet. Work on welfare indicators is still highly incomplete, many have still to be clearly validated and much work is still needed to test the best sampling methods. Studies of horse welfare at different scales have expanded over the last decades and still do. More work, based on a larger array of research groups, and aiming at refining methods, is still highly needed, while comparing rather than opposing welfare assessments between low-income and high-income countries could be quite fruitful. While working conditions in the former may be harsh in terms of climate, equipment and working loads, they may be quite disruptive in the latter too for other reasons (e.g., [74]). Body condition may be poor in low-income countries, but it is far too “good” in high-income countries, and both are equally deleterious for horses’ health (e.g., [16]). Owners may confound apathy with laziness in the former [29], but in the latter, they may also believe that aggressive (suffering) horses may be just bad animals, with the same results in terms of human–horse relationship (e.g., [37]). In both the SEBWAT and AWIN protocols, horses’ responses to humans take a large part, which is important as SEBWAT studies demonstrate a relationship between responses to humans and other behavioral and health measures, and other studies confirm that, beyond reflecting the human–horse relationship, aggressiveness toward humans may reflect chronic or acute pain [20,36,37]. Confronting assessment procedures, without assumptions about outcomes, to make sure we do have the best reflection of their welfare state, i.e., how horses perceive their situation, sounds like a very promising possibility. Claiming that horse welfare is a major issue also in high-income countries may not be what is socially expected, but it is a necessity if we want horse conditions to improve. This is also why it is so important to have undisputable approaches.

### 3.2. Suggestions for Future Improvements of Existing or Future Protocols

#### 3.2.1. Welfare Indicators

A review of animal-based indicators and proposals can be found in Lesimple [1]; thus, we will evoke here only a few examples.

There is an overall lack of indicators of positive welfare. Some new or better-defined indicators may be worth investigating: “alertness”, “attentional engagement” and “responsiveness” are a strong part of the SEBWAT protocol and have revealed interesting [29,39,43], as well as some acoustic signals (e.g., [75]), and would deserve refining. Ear positions during feeding on roughage or grass correlate with cognitive biases, and hence represent horses’ perception of their situation [76] and this criterion is under validation. Burn et al. [29] identified positive reactions to human approaches as a sign of good welfare. This may be the case but will be more or less reliable according to the test performed. Fureix et al. [35] showed that a test with a motionless person did not predict a horse’s reactions in other more “intrusive” tests, such as approaching or touching the animal, a finding also observed by Burn et al. [29]. Unresponsiveness to human approach has been interpreted as “neutral” by Dalla Costa et al. [10,23] and Popescu and Diugan [33], but could also be a sign of apathy. “Depressed” horses react less to human approach and tactile stimulations than non-depressed horses [40]. Only by crossing different measures can we make sure the indicators are valid (e.g., Fureix et al. [40] showed that “depressed” horses had abnormally low levels of cortisol, and Burn et al. [29] showed that they were more at risk to have wounds or anemia). Further investigation is needed on both the meaning of these reactions and the best testing procedure (e.g., “chin test”, “avoidance test”,“sudden approach” ?). Assessments in the home environment have to include well-known abnormal behaviors, such as stereotypies, but also other abnormal repetitive behaviors. Some indicators may be too complex, such as the multicomponent horse grimace scale that different authors mention as being difficult to assess appropriately or too closely related to acute rather than chronic pain [25,49]. Actually, it may be more productive to focus on ear positions with ears backward being associated with all pain scales and being the most easily assessed part of the face [20].

Resource-based indicators, absent from SEBWAT, may be a problem in other protocols. Although there is one publication on stall size and lying behavior [77], there is no clear knowledge of what an “optimal” stall size should be, especially as it is an inappropriate housing from the start (e.g., [70,78]). Should not future protocols indicate that being permanently in a single stall is per se a welfare alteration? Bedding quality versus cleanliness, free exercise versus working exercise, but also door heights (which could lead to back problems, e.g., [59]), availability of semi-continuous feeding, quality of equipment [68] would all deserve further consideration.

#### 3.2.2. Sampling and Scoring: Some Further Thoughts

Discrepancies in the prevalence of welfare problems may be explained by differences in sampling methods and efforts. For example, stereotypic behaviors are assessed in AWIN by observing a horse for a single one-minute period. Lesimple et al. [59] first defined the behaviors concerned: “for a behavior to be considered as SB/ARB, the behavioral sequence had to be repeated at least 3 times successively and observed 5 times, independently of the period of observation”. In their study, the assessor stood motionless at one end or in the middle of lines of stalls so that she could see all the horses. In many cases, the stables were disposed along corridors with a row of stalls on each side. When positioned at the mid-line of the corridor, it was therefore possible to easily see four stalls at a time. The behaviors concerned were scored (in terms of presence/absence) every time they occurred (all occurrences, [17]) over 10-min periods. In all, each horse was observed for 18 h. Although such a long time of observation would not be feasible for large on-farm assessments, one minute is certainly too short. Moreover, being less focused on one single horse may prevent it from being disturbed by the observer’s presence and lower its propensity to perform stereotypic behaviors. Another approach consisted of walking slowly but continuously along the stalls and noting, as in scan sampling, whether the horse was performing or not stereotypic behaviors (e.g., [67]). One further aspect is the necessity to extend and validate the whole variety of abnormal repetitive behaviors that horses produce under restricted conditions.

The timing of observation is important: in order to evaluate whether the pre-feeding period was not only a time when horses perform more stereotypic behaviors (e.g., [50]), but also whether pre-feeding or other day time periods are equally valid for assessing a horse’s propensity to produce them, one trained experimenter (NL) observed respectively 42 and 34 riding school horses of various ages, sexes and breeds, in their stall, following Lesimple et al.’s [59] protocol, both at pre-feeding and outside feeding time: the results show that there is or not a correlation in the frequency of stereotypic behaviors between both time periods according to the facility (rs = 0.44, *p* = 0.004 versus rs = −0.28, *p* = 0.11). A choice must thus be made between one or the other period or systematically both. With this same protocol, we were able to show, despite a small sample, a highly significant stability of the stereotypic behaviors over time when horses remain under the same conditions: comparison of data for observations performed by two different trained observers (MS and EG respectively) made three years apart (2016–2019) of the same nine riding school horses that had remained in the same facility revealed a remarkable stability of individual differences (rs = 0.91, *p* = 0.0005). Similar results were obtained for the number of negative (ears laid back, gazing at the experimenter, approaching her or not) behaviors during human–horse relationship tests (N = 9, rs = 0.83, *p* = 0.005). Such high correlations suggest that repeatability and inter-observer reliability of these measures may be much higher than those observed by Czycholl et al. [48] with AWIN and Burn et al. [29] with SEBWAT, possibly by observing more closely subtle behavioral changes (e.g., ears turning backwards but not pinned) or by changing slightly the test procedure. The stability of stereotypic behaviors in a stable management situation is especially interesting, as changes in practices my lead to rapid changes in the frequency of stereotypic behaviors [78].

High inter-individual reliability could also be verified for two recently proposed indicators: (1) neck shape outside work that two trained observers (NL and EG) assessed in 35 riding-school horses of various ages, sexes and breeds, following Lesimple et al.’s [41] method (kappa = 0.70); and (2) ears backward while feeding on roughage, assessed in 20 of these horses (kappa = 0.95). We also tested the minimum number of measures necessary for obtaining representative data, using 42 riding-school horses: ear positions (forward, backward, sideward or asymmetrical) were recorded whilst horses were foraging on the ground (hay/straw). Observations were performed when the stables were quiet, outside feeding and working times. The experimenter walked slowly and regularly (1 step/second) in the middle of the corridor, or 2 m away from the boxes in stables with one line of boxes. She approached each box slowly, in order to be able to see the ear positions through the trough opening or box door, remaining at a distance. The instantaneous ear position of the feeding horse was silently noted (only if the horse kept feeding and paid no attention to the observer). The observer then resumed her walk along the midline up to the next stall. These samplings were made every day, for three consecutive days, and distributed all along the day until 10 and 20 ear positions were obtained per horse. The percentage of scans in each position was calculated for each horse, and we analyzed the animals’ ranking in relation to the number of data obtained: the results show that 10 measures were as representative as 20 for the ears positions: backward (rs = 0.76, *p* = 2.5e^−0.9^), forward (rs = 0.68, *p* < 0.001) and sideward (rs = 0.43, *p* = 0.003).

### 3.3. Going back to Fundamentals: Behavior, the Window on Welfare State

On one hand, “Welfare Quality^®^” and the associated five freedoms put much emphasis on a combination of animal- and resource- based indicators to draw attention toward freeing animals from negative experiences, especially in the physical sphere. On the other hand, stakeholders are more particularly sensitive to the physical management of horses (e.g., [79]). Current proposed protocols for on-farm assessment, maybe as a consequence, put emphasis on health aspects and overall physical aspects, and welfare assessment has become more and more a veterinarian sphere. Although it has been emphasized for some time that “contentment”, a “mental aspect” and highly subjective experience, is part of well-being [80], cleanliness, hoof regularities and other such aspects may be treated in priority or equally with a horse’s mental state. By putting on the same level their four principles, good housing, good feeding, good health and good behavior, Welfare Quality^®^ promoters have somewhat biased the debate. Good feeding should not just mean being free from hunger but also being able to perform feeding behavior in an appropriate way, i.e., almost continuously (e.g., [81]). Good housing means that horses should be able to express locomotor or social behaviors appropriately, and good health means no pain or physical suffering, which, when altered, is expressed through behavioral modifications. Thus, behavior is a core aspect of welfare, being the interface between the organism and its environment. It is the means through which animals express emotions, try to cope with difficulties or to reach a goal. Any horse which suffers hunger, thirst, discomfort or pain will show behavioral modifications. Because welfare is a subjective experience, similar conditions do not induce the same internal states in different individuals, as they have different physiological and mental characteristics and have had different experiences. This is the rationale behind the preferential use of animal-based indicators rather than resource-based indicators. However, because “good behavior” is a separate section, we still often rely upon our (human-based) appreciation of resources to assess whether a horse is in good housing and/or health conditions.

“Moving beyond the five freedoms” [82] may mean going toward a “provisional” model where contentment is the aim, but it may also mean that we should move back to fundamentals. Because the welfare state is a subjective experience, and because animals, as nonverbal beings, express their internal states through behavior, behavior should be the starting point of welfare assessments. In a non-specific way, a limited array of behavioral modifications can indicate whether the animal is suffering physically or mentally. Horses with compromised welfare, in pain or in distress, can become aggressive, apathetic and unresponsive, and develop abnormal behavior. There is growing evidence that a “content” horse is attentive, and quietly explores, feeds, rests and has calm interactions with humans. Putting back behavior at the core of the assessment means that the first step in the assessment should be to evaluate horse behavior. A horse’s hoofs may be irregular, but that horse is nevertheless “content”, thus not suffering from compromised welfare. Clarifying the validation and description of behavioral or postural indicators in order to improve their recognition (e.g., [6,7,83]) is probably currently the highest priority, so that assessments can start by evaluating whether the horse is “feeling” good. If so, then the measures of other parameters may be of secondary importance; if not, then a thorough examination of the factors (feeding, housing and health) involved could help understand why that horse does not “feel good”. This is an important point in terms of message to laymen, in order to draw attention, develop awareness and provide knowledge on how horse behavior may constitute a very useful window on horses’ internal states. This may mean also returning to more ethological methods and measures, promoting assessor teams including persons trained primarily in ethology.

## 4. Conclusions

As scientists, our responsibility is high in terms of information concerning animal welfare. Contrarily to the predominant cultural representation, horse welfare issues are as important in high-income countries as in low-income countries; they are just different. On-farm welfare assessments in this context have a crucial role to play, especially if they are distributed to the public: they must give an accurate image of the actual problems but also have to help promote good practices and representation of animals in good welfare, i.e., the point to be reached. The domestic situation has to be a situation where there are compromises between the “ideal” situation and economical and spatial constraints. However, in view of the extensive scientific literature on the topic, it is no longer possible to let horse caretakers believe that permanent single-stall housing, amongst other common practices, is associated with good welfare. We have seen here that proposing protocols based on indicators and sampling methods that still need to be refined, leads to under-evaluation and under-representation of the real current welfare problems and their numerical importance. Because welfare is a subjective experience, the true representation of an individual’s actual welfare status has to be measured by objective assessment tools (that are validated and have a scientific basis) and by well-trained observers.
